# Integrated approaches to delivering cancer screenings to address disparities: lessons learned from the evaluation of CDC’s Colorectal Cancer Control Program

**DOI:** 10.1186/s43058-022-00346-7

**Published:** 2022-10-11

**Authors:** Sujha Subramanian, Florence K. L. Tangka, Amy DeGroff, Lisa C. Richardson

**Affiliations:** 1 RTI International, 307 Waverley Oaks Road, Waltham, MA 02452 USA; 2 Division of Cancer Prevention and Control, Centers for Disease Control and Prevention, 4770 Buford Highway, NE, Mailstop S107-4, Atlanta, GA 30341-3717 USA

**Keywords:** Integrated interventions, Cancer screening, Disparities

## Abstract

**Background:**

The Centers for Disease Control and Prevention launched the Colorectal Cancer Control Program to increase colorectal cancer screening among groups with low screening uptake. This engagement has enabled the health systems participating in the program to enhance infrastructure, systems, and process to implement interventions for colorectal cancer screening. These improvements have enabled other health promotion innovations such as the delivery of integrated interventions and supporting activities (referred to as integrated approaches) for multiple cancers. Using implementation science frameworks, the program evaluation team has examined these integrated approaches to capture the experiences of the awardees, health systems, and clinics.

**Methods and results:**

The findings from this comprehensive evaluation are presented in a series of 3 manuscripts. The first manuscript provides a conceptual framework for integrated approaches for cancer screening to support comprehensive evaluations and offers recommendations for future research. The second manuscript presents findings on key factors that support readiness for implementing integrated approaches based on qualitative interviews guided by implementation science constructs. The final manuscript reports on the challenges and benefits of integrated approaches to increase cancer screening in primary care facilities based on lessons learned from three real-world implementation case studies.

**Conclusion:**

Integrated models for implementing cancer screening could offer cost-effective approaches to reduce healthcare disparities. Additional implementation science-based systematic evaluations are needed to ensure integrated approaches are optimized, and cost-efficient models are scaled up.

## Background

The uptake of colorectal cancer screening remains low for populations that experience a disproportionate burden from colorectal cancer incidence and mortality [1]. Racial minorities, rural populations, and low-income individuals are among those who have suboptimal colorectal cancer screening rates [2, 3]. These disparities exist despite guideline-recommended screening tests and the availability of evidence-based interventions and supporting activities to assist health systems to increase screening uptake [4, 5]. These evidence-based interventions include provider assessment and feedback, provider and patient reminders, and reduction of structural barriers as described in the *Guide to Community Preventive Services* [5]. Supporting activities are actions taken to enhance the adoption, implementation, and sustainability of evidence-based interventions and include practice facilitation, technical assistance, and learning collaboratives.

The Centers for Disease Control and Prevention (CDC) implements the Colorectal Cancer Control Program (CRCCP) to increase colorectal cancer screening among people between 45 and 75 years of age (50 and 75 years until 2021) with a focus on populations with low screening uptake. CDC launched the groundwork for initiating the CRCCP by conducting a 4-year colorectal cancer screening pilot program in five sites from 2005 to 2009. Based on the successful implementation and lessons learned from the pilot program, CDC received funding from the US Congress to launch the CRCCP in 2009. For the period from 2009 to 2015, CRCCP supported 20 programs with a focus on colorectal cancer screening promotion for populations experiencing screening disparities along with the delivery of CRC screening and diagnostic services. The next round of funding, from 2015 to 2020, was initiated with the specific goal of implementing evidence-based interventions to increase colorectal cancer screening among groups experiencing disproportionate impact due to low screening uptake [6]. CDC funded 30 CRCCP awardees during this period. In the current iteration of the program, which was initiated in 2020, CRCCP continues to support the implementation of evidence-based interventions with a strong emphasis on increasing diagnostic colonoscopy completion. Currently, the CRCCP funds 35 awardees which include 20 states, 8 universities, 2 tribal organizations, and 5 other types of organizations. The CRCCP has evolved over time to provide support for capacity building and readiness activities to ensure the successful implementation of interventions in health systems where screening uptake is low [7]. This engagement with the CRCCP has allowed these health systems to enhance infrastructure and systems to implement interventions for colorectal cancer screening, and these improvements have also been leveraged for other health promotion activities.

The support provided by the CRCCP has enabled health systems such as federally qualified health centers to engage in innovations. One innovative approach that these health systems are implementing is to deliver integrated interventions and supporting activities for colorectal, breast, and cervical cancer, when appropriate. Integrated interventions are defined as the joint delivery of evidence-based interventions for multiple cancer screenings, for example, when health systems implement joint patient reminders through phone calls or via mailings for patients who are due for more than one cancer screening. Similarly, integrated supporting activities could include practice facilitation to optimize electronic medical records for more than one cancer screening.

Integrated interventions and supporting activities (referred to as integrated approaches) can offer important insights to guide implementation approaches to address cancer screening disparities faced by populations receiving care at health systems that serve groups that have been economically or socially marginalized. First, integrated approaches can offer a patient-centered approach by allowing for single-visit screenings for multiple cancers that can reduce the number of visits and time spent undergoing cancer screenings. Second, integrated approaches can provide synergies and efficiencies to enable health systems that are resource-constrained to sustain evidence-based interventions. Using implementation science frameworks and qualitative and quantitative data collection, CDC’s evaluation team has examined these integrated approaches to capture the experiences of the awardees, health systems, and clinics. In the accompanying series of papers, we present early lessons from evaluations of integrated approaches across selected CRCCP awardees to guide future systematic assessments.

## Overview of CRCCP manuscript series 

In the first manuscript [8], we present a conceptual framework to describe the integrated cancer screening processes and to support the evaluation of integrated approaches. This framework was developed through consultations with CRCCP awardees, health systems, and partners, such as primary care associations and community advocacy organizations, to ensure real-world applicability. The authors provide examples of integrated interventions at the individual, provider, health system, program, and community levels. For example, multiple cancers can be targeted at the individual level for patient reminders and at the providers’ level with alerts through flags in the electronic medical record system. Furthermore, at the program level, blended funding across colorectal, breast, and cervical cancers can be provided to health systems. In the community, small media can be designed to address all three cancers instead of each one individually. Although integrated approaches can be efficient, there were challenges caused by differing eligibility for screenings by age, gender, frequency, and location of services. Awardees ranked complexity, cost, implementation climate, and engagement of appropriate staff in the intervention implementation process among the most important factors to implement interventions for cancer screenings successfully. This manuscript also highlights measurement challenges and recommends future research areas to address current gaps in the literature on integrated interventions and screenings.

The second manuscript [9] reports on key factors that support readiness for implementing integrated evidence-based interventions and supporting activities. The CRCCP evaluation team identified 4 overarching factors that contribute to clinic readiness for delivering integrated interventions and supporting activities: the funding environment, clinic governance structure, information sharing within clinics, and clinic leadership support. Implementers, including state health departments, support clinic teams’ readiness for integrated implementation by providing coordinated application processes and combined funding streams and by funding other organizations, such as primary care associations, to provide technical assistance to support the efficient implementation of integrated interventions and strategies into existing clinic workflows. Features of both the external context and internal clinic organization can support readiness to implement integrated approaches to delivery screenings for multiple cancers.

The third manuscript (Tangka et. al.: Improving the efficiency of integrated cancer screening delivery across multiple cancers: case studies from Idaho, Rhode Island, and Nebraska, under review) uses a case study approach to identify the challenges and benefits of integrated approaches to increase cancer screening in primary care facilities. In Idaho, the results from the checklist-based planning review revealed areas that organizations should enhance before they implement integrated interventions and identified challenges, including lack of capacity, limited staff availability, and staff turnover. In Rhode Island, patient navigation activities were initially focused on colorectal cancer screening but evolved to include breast and cervical cancer screenings as well. Implementing integrated patient navigation with discussions around multiple cancer screenings was found to be an efficient approach, but patients were not always willing to discuss all cancer screenings they were eligible to receive. Nebraska implemented an integrated payment approach to create a more sustainable and efficient model. The state health department changed its payment system from fee-for-service to fixed-cost subawards with its local health departments, which integrated funding for multiple cancer screenings. Screening uptake improved for breast and cervical cancer but was mixed for colorectal cancer screening. Future assessments will be conducted to evaluate external and internal factors that affect screening uptake across different cancers in integrated screening models.

## Conclusion

In this series of 3 papers, we provide an early assessment of the implementation of integrated interventions and supporting activities for cancer screening, and future research can build on these findings to identify optimal implementation models. A key facilitator for the integrated approaches identified in this series is leadership support at the awardee and health system levels which allow for blended funding streams and prioritization of coordination across multiple cancer screenings. Additionally, health systems that are already implementing the same evidence-based interventions separately for each type of cancer screening, for example, provider assessment and feedback for colorectal, breast, and cervical cancer screening, are better positioned to more rapidly integrate interventions. Although most health systems will likely be able to streamline interventions across multiple cancer screenings, challenges related to capacity, resources, and organizational structure remain barriers to successfully implement integrated evidence-based interventions and supporting strategies.

In this current series of papers, we focus on multiple cancer screenings, but CRCCP awardees and health systems are also testing approaches to integrate cancer screenings with services for other chronic diseases, including hypertension screening and diabetes care. CDC administers several chronic disease programs in addition to the CRCCP, and these include the National Breast and Cervical Cancer Early Detection Program (NBCCEDP) and the **W**ell-**I**ntegrated **S**creening and **E**valuation for **WOM**en **A**cross the **N**ation (WISEWOMAN) program. All these programs provide funding to implement evidence-based interventions and supporting strategies. These programs are also often implemented by the same set of awardees and health systems which provides an ideal environment for delivering and evaluating integrated approaches. Implementation science-based systematic evaluations can ensure that these integrated approaches are optimized and cost-efficient models are scaled up. These integrated models could offer cost-effective approaches to reduce healthcare disparities. Additionally, learnings across disease categories can help accelerate progress by rapidly diffusing effective innovations.

## Data Availability

The underlying data generated during the study are not publicly available as health system participants cannot be adequately masked, and only summarized data was available to the evaluation team. Data were collected by RTI International under contract with the Centers for Disease Control and Prevention.

## Abbreviations

CDC: Centers for Disease Control and Prevention
CRCCP: Colorectal Cancer Control Program
